# Supplementary material for: Transcriptome analysis of bread wheat leaves in response to salt stress
Source: PLoS One. 2021 Jul 9;16(7):e0254189. doi: 10.1371/journal.pone.0254189 (PMC8270127; doi:10.1371/journal.pone.0254189)
Supplement: S2 Fig — (DOCX) [file pone.0254189.s002.docx]

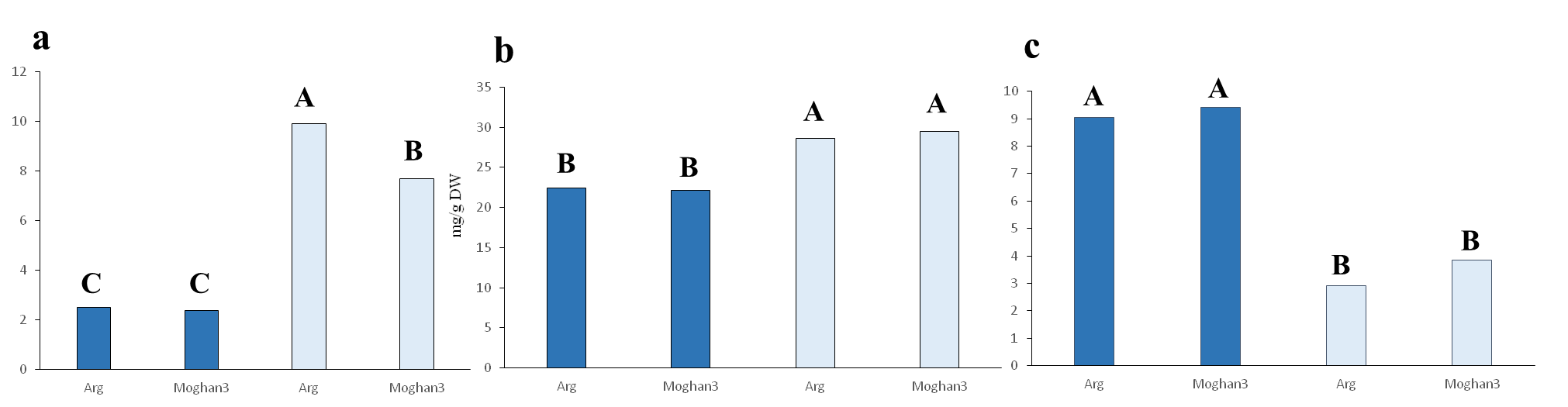


S2 Fig. Comparison of means for changes in Na^+^ content (a), K^+^ content (b) and K^+^/Na^+^ ratio (c) in the roots of Arg and Moghan3 under the normal and salinity treated conditions at the probability level of 5%. Dark blue and light blue bars relates to the normal and salt-stressed conditions, respectively.
