# Supplementary material for: Transcriptome analysis of bread wheat leaves in response to salt stress
Source: PLoS One. 2021 Jul 9;16(7):e0254189. doi: 10.1371/journal.pone.0254189 (PMC8270127; doi:10.1371/journal.pone.0254189)
Supplement: S3 Fig — (a) Out of 4290 DEGs, 110 and 98 genes were exclusively expressed under the salt stress (STL) and control (CL) conditions, respectively (b) Fold change distribution of 4082 DEGs present in both normal and salt treated samples. (DOCX) [file pone.0254189.s003.docx]

**
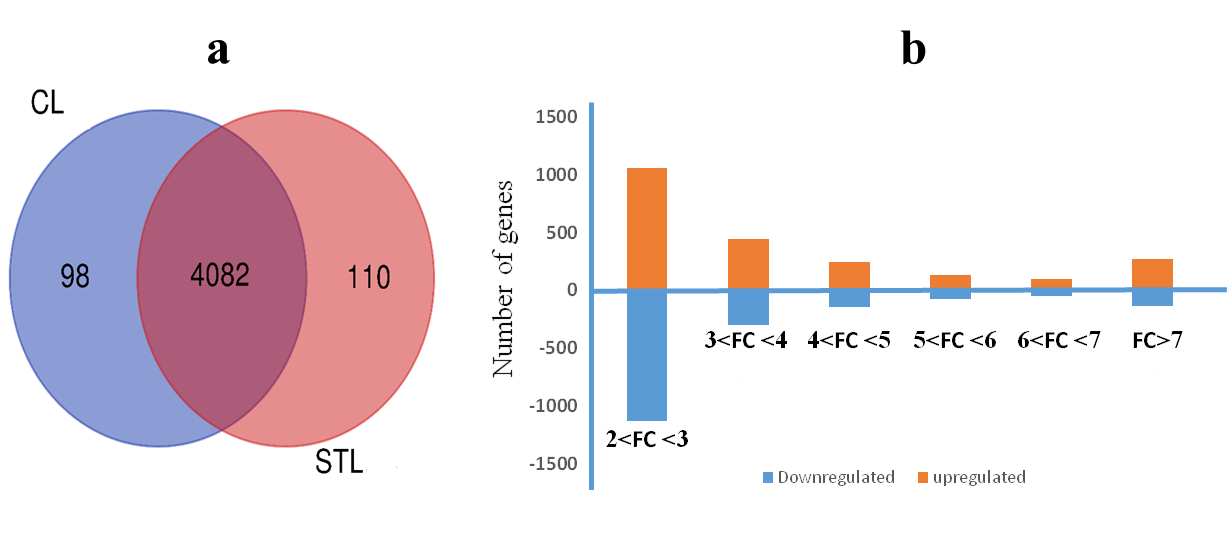
**

**S3 Fig. Survey of the DEGs observed between the control and salt treated samples (Cut off p-value: 0.01). (a) Out of 4290 DEGs, 110 and 98 genes were exclusively expressed under the salt stress (STL) and control (CL) conditions, respectively (b) Fold change distribution of 4082 DEGs present in both normal and salt treated samples.**
