# Supplementary material for: Transcriptome analysis of bread wheat leaves in response to salt stress
Source: PLoS One. 2021 Jul 9;16(7):e0254189. doi: 10.1371/journal.pone.0254189 (PMC8270127; doi:10.1371/journal.pone.0254189)
Supplement: S4 Fig — (a) Annotation statistics of the novel DEGs. (b) GO classification of the novel DEGs under salt stress. (DOCX) [file pone.0254189.s004.docx]

S4 Fig. (a) Annotation statistics of the novel DEGs. (b) GO classification of the novel DEGs under salt stress


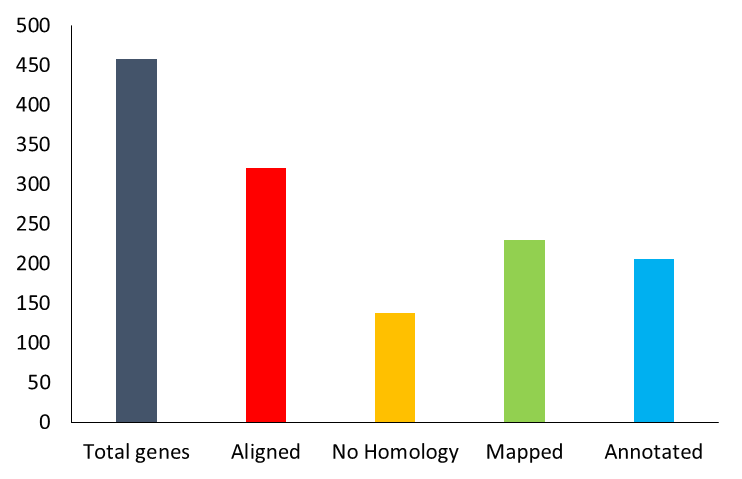

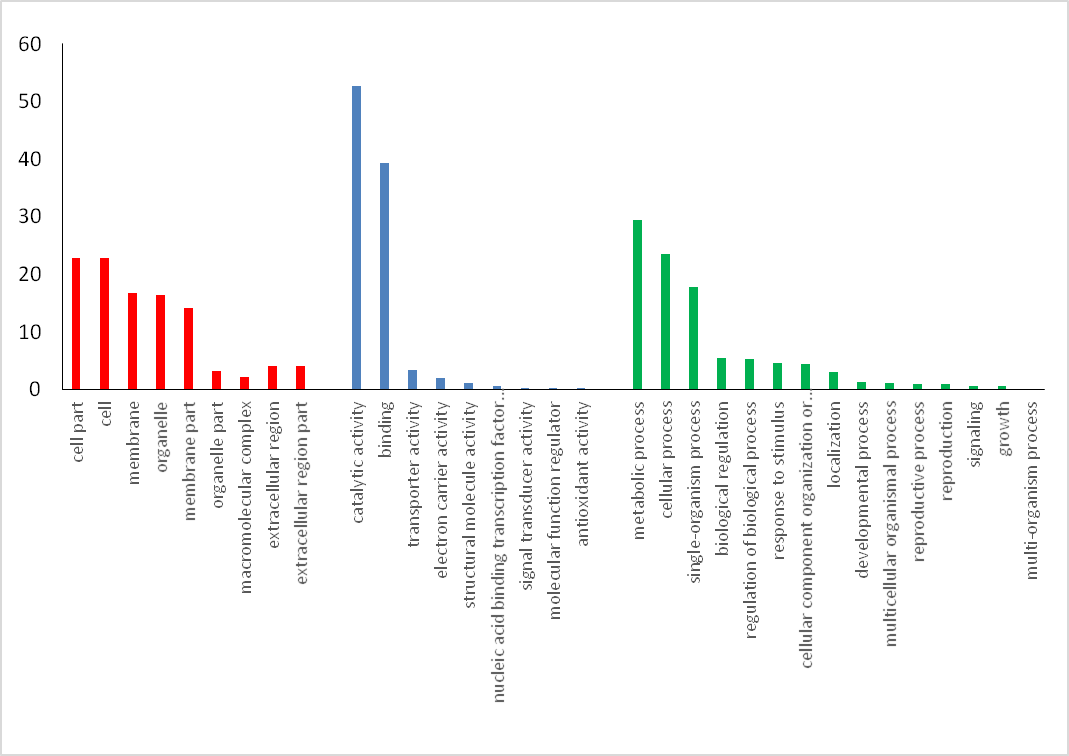


Number of genes

Percentage of transcripts

**(b)**

**(a)**
