# Supplementary material for: Transcriptome analysis of bread wheat leaves in response to salt stress
Source: PLoS One. 2021 Jul 9;16(7):e0254189. doi: 10.1371/journal.pone.0254189 (PMC8270127; doi:10.1371/journal.pone.0254189)
Supplement: S5 Fig — Blue: up-regulated genes and red: down-regulated genes. (DOCX) [file pone.0254189.s005.docx]

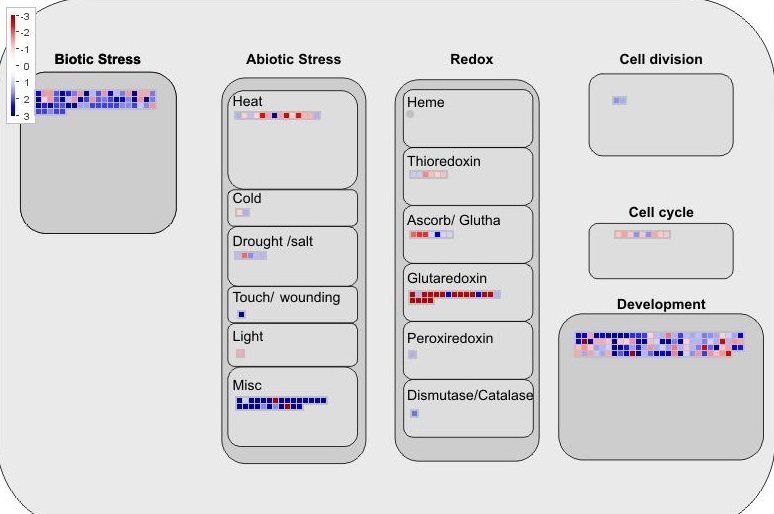


S5 Fig. Cellular pathway overview of the DEGs in *T.aestivum* under salinity stress using Mapman. blue: up-regulated genes and red: down-regulated genes.
