# Supplementary material for: Transcriptome analysis of bread wheat leaves in response to salt stress
Source: PLoS One. 2021 Jul 9;16(7):e0254189. doi: 10.1371/journal.pone.0254189 (PMC8270127; doi:10.1371/journal.pone.0254189)
Supplement: Attachment — Submitted filename: Comments.docx [file pone.0254189.s018.docx]

Dear Editorial bard

Authors studied two varieties of wheat plant under salt stress using physiological and molecular (deep sequencing and qRT-PCR) aspects. The studied varieties are extreme considering their tolerance to salinity. They found several differentially expressed genes in salt tolerance variety. Enriched biological pathways of stress responsive genes are studied in the current study. Finally, authors confirmed deep sequencing outputs using real-time PCR. There are some comments that authors should consider:

1. Why deep transcriptome sequencing not studied for Moghan variety while evaluated physiologically and also by real-time PCR.
2. Please describe in the material and methods that which variety is utilized for deep sequencing.
3. It is not described in the material and methods that how Na and K content are measured.
4. Please clarify in the in the material and methods that how you found significant DEGs in real-time PCR.
5. It is not clear why Actin is used as an internal control gene. Dose it previously used for salt stress in wheat or other plants? Reference is required.
6. TopHat software description should be explained in material and methods instead of results (page 8 )
7. Are you sure that mentioned Gene Ensembl Id (column 2 of Table S1) are belonged to Ensembl? I could not found them at Ensembl.
8. It is not clarified in the S2, S3 and S4 tables that which GOs are enriched in salinity or normal conditions.
9. Gene Accession Id is necessary for S2, S3 and S4 tables. Please add this column.
10. Column 1 of Table S10 is not clear. If sequences are aligned after blast use “aligned” term instead of “Blasted”.
11. Some values of log(Fc) of table S10 are obscure. Please check rows 25, 47, 93 and … . likely some values of description column are not clear ([Hordeum vulgare vulgare], ---NA---).
12. Ven diagram is not appropriate choice for showing DEGs in Fig S3(a). if you are insisted on showing your data using Ven diagram then remove “4082”. But it is better to use another type of graphs.
13. Fig S3(b) is ambiguous for common values. Use > or < signs along with values.
14. Please use the correct terms instead of “Blasted” and “No Blast” in Fig S4A. Additionally, terms of manuscript (page 11, paragraph 1) are not coincided with FigS4A.
