# Supplementary material for: Transcriptome analysis of bread wheat leaves in response to salt stress
Source: PLoS One. 2021 Jul 9;16(7):e0254189. doi: 10.1371/journal.pone.0254189 (PMC8270127; doi:10.1371/journal.pone.0254189)
Supplement: Attachment — Submitted filename: COMMENTS FOR THE AUTHOR.docx [file pone.0254189.s019.docx]

COMMENTS FOR THE AUTHOR:

Transcriptome analysis of bread wheat leaves in response to salt stress

The manuscript is well written and presents new insights regarding the wheat leaves transcriptome exposed to salinity. Minor corrections are suggested.

In introduction, wheat transcriptome analysis of other plant tissues in salinity besides leaves can also be cited to make it comprehensive. Abbreviations, when used for the first time in text, should be given in brackets with the complete forms. A few suggestions are given as under.

Page 2, line 16: The word “Soil” may be removed. It is better to begin the sentence with salinity.

Page 3, line 37: The first paragraph of “Introduction” needs improvement due to the repetition of words like yield and salinity.
Page 9, line 196-199: Revise the statement.

In figures, please use an appropriate font size to make the text more visible especially the X and Y axis in curves are not clear.
